# Effect of simultaneous integrated boost concepts on photoneutron and distant out-of-field doses in VMAT for prostate cancer

**DOI:** 10.1007/s00066-023-02138-x

**Published:** 2023-09-14

**Authors:** Benjamin Gauter-Fleckenstein, Sebastian Schönig, Lena Mertens, Hans Oppitz, Kerstin Siebenlist, Michael Ehmann, Jens Fleckenstein

**Affiliations:** Department of Radiation Oncology, University Medical Center Mannheim, University of Heidelberg, Theodor-Kutzer Ufer 1–3, 68167 Mannheim, Germany

**Keywords:** Simultaneous integrated boost, Flattening filter free, Volumetric modulated arc therapy, Prostate carcinoma, Scatter radiation dose, Photoneutrons

## Abstract

**Background:**

A simultaneous integrated boost (SIB) may result in increased out-of-field (D_OOF_) and photoneutron (H_PN_) doses in volumetric modulated arc therapy (VMAT) for prostate cancer (PCA). This work therefore aimed to compare D_OOF_ and H_PN_ in flattened (FLAT) and flattening filter-free (FFF) 6‑MV and 10-MV VMAT treatment plans with and without SIB.

**Methods:**

Eight groups of 30 VMAT plans for PCA with 6 MV or 10 MV, with or without FF and with uniform (2 Gy) or SIB target dose (2.5/3.0 Gy) prescriptions (CONV, SIB), were generated. All 240 plans were delivered on a slab-phantom and compared with respect to measured D_OOF_ and H_PN_ in 61.8 cm distance from the isocenter. The 6‑ and 10-MV flattened VMAT plans with conventional fractionation (6- and 10-MV FLAT CONV) served as standard reference groups. Doses were analyzed as a function of delivered monitor units (MU) and weighted equivalent square field size A_eq_. Pearson’s correlation coefficients between the presented quantities were determined.

**Results:**

The SIB plans resulted in decreased H_PN_ over an entire prostate RT treatment course (10-MV SIB vs. CONV −38.2%). Omission of the flattening filter yielded less H_PN_ (10-MV CONV −17.2%; 10-MV SIB −22.5%). The SIB decreased D_OOF_ likewise by 39% for all given scenarios, while the FFF mode reduced D_OOF_ on average by 60%. A strong Pearson correlation was found between MU and H_PN_ (*r* > 0.9) as well as D_OOF_ (0.7 < *r* < 0.9).

**Conclusion:**

For a complete treatment, SIB reduces both photoneutron and OOF doses to almost the same extent as FFF deliveries. It is recommended to apply moderately hypofractionated 6‑MV SIB FFF-VMAT when considering photoneutron or OOF doses.

## Introduction

Recently, moderately hypofractionated intensity modulated radiotherapy (IMRT) regimens were assigned as standard of care in interdisciplinary national and international guidelines [1, 2] for prostate cancer treatments. We are currently faced with an increasing number of simultaneous integrated boost (SIB) concepts being used both in clinical trials and in daily routine (clinicaltrials.gov yielded 85 active or recruiting trials for “simultaneous integrated boost”). Evidence for these approaches is drawn from moderate hypofractionation trials for prostate [3–8]. For these approaches, an SIB concept is often the method of choice. The SIB increases fractional radiation dose in selected regions of the treated volume. This leads to shortening of overall treatment time and results in steeper dose gradients toward more radiosensitive organs and consequently a higher dose conformity [9]. Furthermore, moderately increased fractional doses potentially result in beneficial fractionation effects in tumors with low alpha/beta values.

High-energy photons may, depending on their energy, generate photoneutrons (PN) when interacting with beam-shaping components in the medical linear accelerator (linac) treatment head. The primary PN-generating structures were identified [10] to be the radiation target, primary collimator, flattening filter, multileaf collimator (MLC), and jaws. The generated PN typically possess kinetic energies between 0.2 and 2.5 MeV. To account for the higher radiobiological effectiveness (RBE) of neutron radiation, the physical dose D in Gray (Gy) is converted into the equivalent dose H in Sievert (Sv) by multiplication of D with a weighting factor, which is between 2.5 and 20.7 (ICRP 103, [11]). Due to the increased RBE, PN have undesired biological and physical effects on surrounding healthy tissue but also on implanted electronic devices such as cardiac pacemakers and cardioverter-defibrillators, insulin pumps, or neural stimulators (AAPM158, [12]).

With respect to radiation protection, it is furthermore relevant to note that matter within the treatment beam leads to formation of scattered radiation that, in combination with radiation transmitted through the beam-shaping devices, leads to distant out-of-field dose (D_OOF_) in the patient. The OOF radiation contributes to potentially unmonitored and unaccounted radiation exposure at a distance from the treated volume, which means there is an increased risk for secondary cancers in organs distant to the primary cancer region [13]. This dose remains unmonitored because not all treatment planning systems (TPS) explicitly account for distant scattered radiation [14] and if they do, CT scans typically do not exceed the adjacent body regions closest to the target volume.

In conventional three-dimensional conformal radiation therapy, the flattening filter (FF) generates a uniform photon fluence within the therapeutic beam. Here, up to 40 × 40 cm^2^ uniform photon beams are shaped through position-dependent beam attenuation into a conformal radiation field. In intensity modulated radiation therapy (IMRT), several smaller segments of irregular shaped fields and with differing photon fluences are directed from several angles to superimpose to a uniform dose with high conformity to the treatment volume. Modern IMRT can be applied with continuously moving multileaf collimator (MLC) leaves and jaws during continuously changing gantry positions and varying dose rates (e.g., VMAT [volumetric modulated arc therapy]). While a wedge becomes unnecessary in modulated segmented radiation fields, the FF frequently remains in the beam. Removal of the FF allows for high dose rates (15–25 Gy/min) that are beneficial for irradiation of small and potentially moving volumes with tight margins as present in stereotactic body radiotherapy at the cost of a uniform lateral target fluence profile. Additionally, removal of the FF in IMRT reduces matter in the beam, hence reducing the contamination with PN and also scattered radiation at a distance from the treated volume [13, 15–17].

All the aforementioned modifications (SIB, FFF) result in varying modulation depths (number of required monitor units [MU] divided by prescribed dose to target) and differing average equivalent square field sizes (A_eq_) describing MLC activity. This might influence PN generation and D_OOF_. For locations distant to the isocenter, no studies exist that describe the effect of a prostate SIB in combination with FFF-VMAT in view of neutron contamination or scattered radiation. This might be significant especially for implanted electronic devices in this distance from radiation volumes such as cardiac implanted electronic devices (CIEDs; [18, 19]). In parallel to the increasing use for SIB concepts, there is currently an ongoing trend toward hypofractionation in general, and for prostate radiation therapy (RT) in particular. We therefore addressed the question of whether this trend is accompanied by an increase in out-of-field doses originating from either scattered photons or photoneutrons. In this context, we investigated the effect of an SIB concept in flattened and flattening-filter free VMAT with 6 and 10 MV for prostate cancer RT on distant neutron- and out-of-field dose in VMAT plans of comparable dose distributions and comparable therapeutic efficacy. The results of this study might influence the decision process in whether RT for prostate carcinoma should be moderately hypofractionated, especially in CIED-bearing individuals, or not.

## Methods

### Treatment sequence generation

Based on the anonymized treatment planning CT data of 30 patients previously treated for prostate cancer, volumetric modulated arc therapy treatment plans (all 360° dual arcs) were generated in the TPS (Monaco 5.51, Elekta AB, Stockholm, Sweden). Treatment plans for two different dose prescription approaches were created:

- A uniform dose-to-target concept (CONV): planning target volume (PTV) was prostate and seminal vesicles plus 10 mm isotropic margin (PTV); 37 fractions (fx) with fractional prescription dose D_50%_(PTV) = 2.0 Gy uniformly to entire PTV with a total dose prescription of 74 Gy.
- A simultaneously integrated prostate-boost concept (SIB) in analogy to CHHiP protocol [3]: same PTV as in CONV but with a SIB to the prostate only; 20 fx with fractional prescription dose D_50%_(PTV) = 2.5 Gy and D_50%_(prostate) = 3.0 Gy and a total dose of 50 Gy (PTV) and 60 Gy (SIB).

In total, eight different clinically acceptable and dosimetrically similar treatment plans for all permutations of the following parameters were generated (Table 1):

- CONV or SIB dose prescription to the target volume(s)
- Nominal acceleration potential (6 MV or 10 MV)
- With or without flattening filter (FLAT, FFF)

**Table 1:** Volumetric modulated arc therapy treatment plans^a^ (all 360° dual arcs) for two different dose prescription approaches generated in a 2 × 2 × 2 factorial design: SIB 60 Gy vs. CONV 74 Gy, FLAT vs. FFF, 6 MV vs. 10 MV

Treatment sequence generation				
Energy	Filter position: FLAT		Filter position: FFF	
	Dose concept: CONV	Dose concept: SIB	Dose concept: CONV	Dose concept: SIB
6 MV	6‑MV FLAT CONV (*n* = 30)	6‑MV FLAT SIB (*n* = 30)	6‑MV FFF CONV (*n* = 30)	6‑MV FFF SIB (*n* = 30)
10 MV	10-MV FLAT CONV (*n* = 30)	10-MV FLAT SIB (*n* = 30)	10-MV FFF CONV (*n* = 30)	10-MV FLAT SIB (*n* = 30)

^a^Based on anonymized treatment planning CT data of 30 patients previously treated for prostate cancer

The resulting total number of MU per treatment and the weighted equivalent square field size (A_eq_) as a surrogate for MLC activity of the generated treatment plans are presented in Table 2.

**Table 2:** Prescription doses per fraction (fx) and whole treatment (tx), monitor units (MU), and equivalent square field sizes (Aeq) per treatment as well as average neutron-equivalent (H_PN_) and out-of-field doses (D_OOF_) per MU and treatment fx^a^ for all scenarios

Nom. acc. potential Delivery type PTV coverage	6 MV				10 MV			
	FLAT		FFF		FLAT		FFF	
	CONV	SIB	CONV	SIB	CONV	SIB	CONV	SIB
Prescription dose, fx (Gy)	2	2.5/3	2	2.5/3	2	2.5/3	2	2.5/3
Total dose, tx (Gy)	74	50/60	74	50/60	74	50/60	74	50/60
Monitor units (MU)	839 ± 116	1006 ± 205	962 ± 124	1098 ± 265	717 ± 84	832 ± 137	968 ± 188	1063 ± 209
A_eq_ (cm)	3.0 ± 0.3	2.9 ± 0.5	2.8 ± 0.3	2.9 ± 0.5	3.0 ± 0.3	2.9 ± 0.5	2.7 ± 0.4	2.7 ± 0.5
H_PN_/MU (nSv)	14.9 ± 0.8	15.0 ± 0.6	26.5 ± 0.6	27.0 ± 0.8	494.4 ± 11.1	503.3 ± 13.4	303.1 ± 13.1	305.0 ± 6.1
H_PN_/fx (µSv)	12.5 ± 1.8	15.1 ± 3.1	25.5 ± 3.5	29.7 ± 7.6	354.7 ± 44.2	419.3 ± 72.5	293.8 ± 60.7	324.8 ± 67.3
H_PN_/tx (mSv)	0.5 ± 0.1	0.3 ± 0.1	0.9 ± 0.1	0.6 ± 0.2	13.1 ± 1.5	8.4 ± 1.5	10.9 ± 2.1	6.5 ± 1.4
D_OOF_/MU (µGy)	1.7 ± 0.1	1.6 ± 0.1	0.7 ± 0.1	0.7 ± 0.1	2.3 ± 0.3	2.3 ± 0.2	0.7 ± 0.1	0.7 ± 0.1
D_OOF_/fx (mGy)	1.6 ± 0.3	1.9 ± 0.5	0.7 ± 0.1	0.7 ± 0.2	1.8 ± 0.4	2.0 ± 0.4	0.7 ± 0.2	0.8 ± 0.2
D_OOF_/tx (mGy)	59.2 ± 11.1	38.0 ± 10.0	25.9 ± 3.7	14.0 ± 4.0	66.6 ± 14.8	40.0 ± 8.0	25.9 ± 7.4	16.0 ± 4.0
*r* (MU, H_PN_)	0.93	0.98	0.99	0.99	0.99	0.99	0.98	1
*r* (MU, D_OOF_)	0.84	0.92	0.68	0.73	0.75	0.90	0.78	0.76
*r *(Aeq, H_PN_)	−0.75	−0.71	−0.74	−0.71	−0.55	−0.73	−0.83	−0.72
*r* (Aeq, D_OOF_)	−0.53	−0.66	−0.31	−0.53	−0.17	−0.65	−0.51	−0.49

^a^Mean values and standard deviations, *r* Pearson’s correlation coefficient

A_eq_ is defined as

$$A_{eq}={\sum }_{i=1}^{n}\omega _{i}A_{i{,}eq}$$

where ω_i_ is the fraction of MUs of the i‑th control point and A_i,eq_ is the corresponding control point equivalent square field size. A_i,eq_ is obtained by the ratio of control point area over control point perimeter.

### Measurement set-up and data analysis

**Fig. 1:**
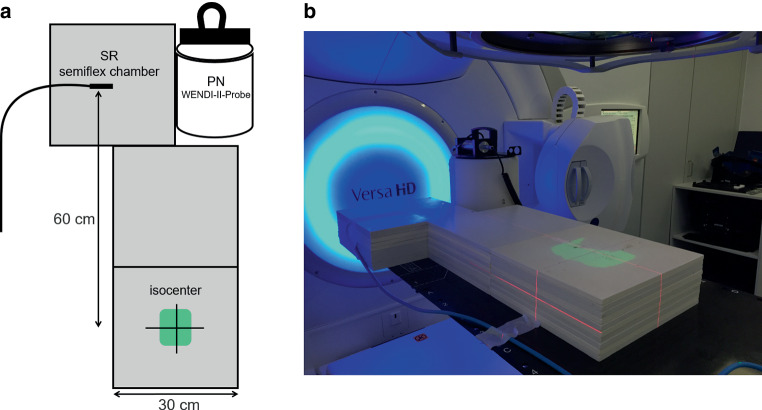
Experimental set-up. Sketch (**a**) and photograph (**b**) of the experimental measurement set-up: 10-cm stacks of solid water equivalent phantom and a neutron detector (H_PN_) as well as an ionization chamber for the measurement of scattered radiation (D_OOF_). Both detectors are located in 61.8 cm distance from the isocenter

Treatment sequences were delivered on a linac (Versa HD, Elekta AB, Stockholm, Sweden) to an anthropomorphous slab phantom arrangement, consisting of three 30 × 30 × 10 cm^3^ stacks of water equivalent phantom (RW3, PTW, Freiburg, Germany) and a neutron detector. The beam isocenter was located in the “pelvic area” at the center of the lowest RW3 stack. The center positions of each detector and the isocenter were 61.8 cm away from each other. See measurement set-up in Fig. 1.

For each VMAT sequence, PN-dose H_PN_ and out-of-field-dose D_OOF_ measurements were performed simultaneously. Out-of-field-dose, comprising scattered and beam-limiting device-transmitted radiation, was measured using an ionization chamber (0.125 cm^3^ semiflex, 31010, PTW, Freiburg, Germany) with photon energy response uncertainty above 50 kV of less than ±5% (useful range 140 kV and above radiation quality), connected to a Unidos Webline electrometer (PTW, Freiburg, Germany). The ionization chamber was placed at the center of the uppermost slab of the slab phantom on the opposite side of the neutron detector after calibration to ambient temperature and pressure. Since one slab of the RW3 phantom has a thickness of 1 cm and no bolus material was used, the depth of the ionization chamber was located at a depth of 0.5 cm below the surface. This represents a typical implantation depth for CIEDs in patients.

The wide energy neutron detection instrument (WENDI‑2 FHT 762 with FH 40 G survey meter, Thermo Fisher Scientific, Erlangen, Germany) is a He3-filled proportional counter with a thermal to 5 GeV energy range according to H*(10), ICRP 74 [20] and a measuring range from 0.01 μSv/h to 100 mSv/h. Its reported sensitivity is 0.84 cps/μSv/h Cf-252 and the device has proven to produce reliable dose readings [21]. Measurement mode was set to “neutron dose only”. Internal software-based calibration procedures according to the manufacturer’s specifications including detection of contaminating X‑ray scattered radiation were respected.

The resulting D_OOF_ and H_PN_ were plotted against two characteristic delivery sequence-defining parameters MU and A_eq_. Subsequently, absolute values for D_OOF_ in Gray and H_PN_ in Sievert were put in relation to the delivered MUs (D_OOF_/MU, H_PN_/MU) to compare values between different dose levels with and without SIB. Measurements were performed once for all 30 plans. To determine the measurement reproducibility, measurements for three representative treatment plans were repeated three times for all eight treatment scenarios. Standard deviation is reported as percent of the corresponding mean value.

The values reported in Table 2 were generated by first determining the relevant quantity (e.g., H_PN_/MU) for each treatment sequence and subsequently generating the mean and standard deviations of the 30 treatment sequences that belong to one treatment approach.

For a comparison of total PN or out-of-field dose resulting from an entire RT course for prostate cancer with the investigated VMAT plans, summed absolute values for all treatment fractions for CONV (total dose 74 Gy; 37 fx) and SIB (total dose 50/60 Gy; 20 fx) were compared. The 6‑ and 10-MV flattened VMAT plans with conventional fractionation (6- and 10-MV FLAT CONV) served as standard reference groups. Using SAS software (release 9.4, SAS Institute Inc., Cary, NC, USA), a linear mixed model was generated to control interaction effects in our 2 × 2 × 2 model. After investigating single effects of photon energy level (6 and 10 MV), position of the flattening filter (FLAT, FFF), and dose prescription (SIB, CONV), a three-way ANOVA was used to investigate the difference in dose reductions between the most promising plan setting in comparison to the standard reference groups. Fixed factors were FF, photon energy, and homogeneity. Random factor was the plan ID. For comparisons between reference plan settings and plan settings with least H_PN_ and D_OOF_, paired *t* tests were used. In general, *p* < 0.05 was considered significant. The F values describe effect sizes.

Finally, Pearson’s correlation coefficients between D_OOF_ and H_PN_ and the aforementioned variables MU and A_eq_ were generated. Correlation between parameters was considered strong for *r* values between 1 and 0.7, moderate for *r* values between 0.7 and 0.3, and weak for *r* values between 0.3 and 0.

## Results

**Fig. 2:**
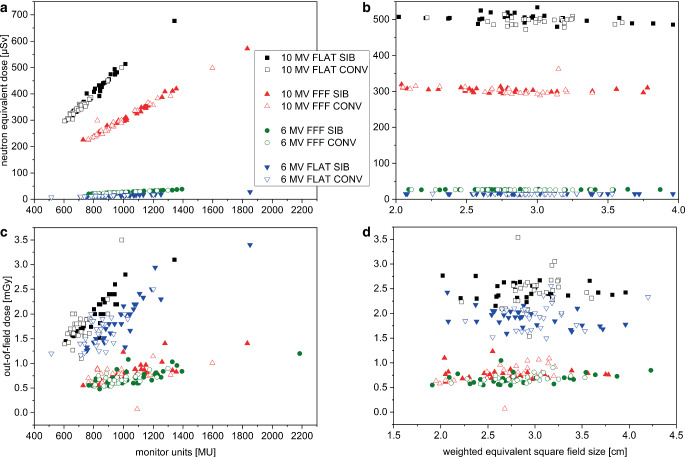
Neutron-equivalent doses and out-of-field doses. Neutron-equivalent dose (**a** and **b**) and out-of-field dose (**c**and **d**) as a function of monitor units per treatment sequence (**a** and **c**) and weighed equivalent square field size (**b** and **d**)

**Fig. 3:**
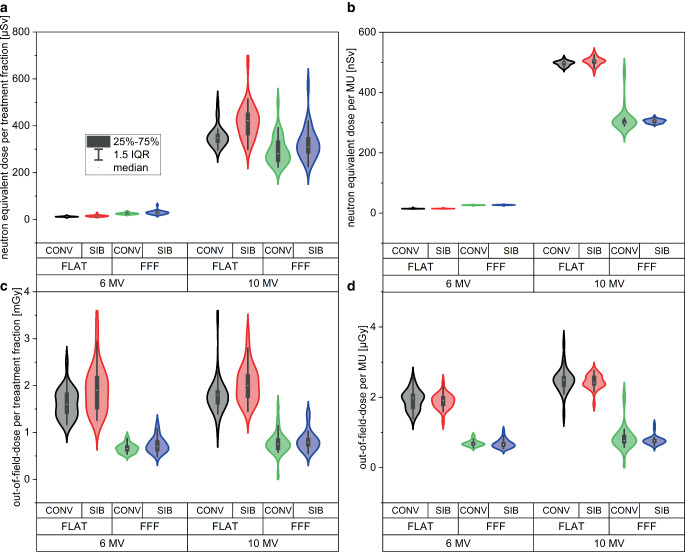
Violin and box plots of the30 treatment plans for each of the eight treatment approaches. Neutron-equivalent doses per treatment fraction (**a**) and per monitor unit (**b**) as well as out-of-field doses per treatment fraction (**c**) and per monitor unit (**d**). *Box plots* indicate one interquartile range (*IQR*)

The resulting neutron-equivalent doses H_PN_ per delivered treatment fraction as a function of either MUs per treatment plan (Fig. 2a) or weighted equivalent square field size (Fig. 2b) are presented in Fig. 2. In analogy, the resulting out-of-field doses D_OOF_ are presented in Fig. 2c and d. In all panels, the eight different treatment scenarios with all 30 treatment sequences are displayed. Mean values are displayed in Fig. 2 while the standard deviations as percent of the corresponding mean value were σ(H_PN_) = 0.80% (between 0.44 and 1.47% for all groups) and σ(D_OOF_) = 0.54% (between 0.26 and 0.91% for all groups). Figure 3 shows violin and box plots of the 30 treatment plans for each of the eight treatment approaches. Neutron-equivalent doses per treatment fraction (panel a) and per MU (panel b) as well as out-of-field doses per treatment fraction (panel c) and per MU (panel d) are displayed. Boxplots indicate one interquartile range (IQR).

The prescription dose, the total dose, the total number of MUs, and the equivalent square field sizes as well as average neutron-equivalent (H_PN_) and out-of-field doses (D_OOF_) per MU and treatment fraction are presented in Table 2 as mean values and standard deviations. Comparing all respective plans together, it becomes apparent that over all of the different scenarios, SIB resulted only in 57 plans in more MUs but actually in another 63 plans in less MUs than their CONV counterparts. Furthermore, the resulting neutron-equivalent—and out-of-field doses per MU and per complete treatment as well as the correlation coefficients are displayed.

### Neutron-equivalent dose between SIB and CONV plans with and without flattening filter, as a function of monitor units, and equivalent square field size

As expected, flattened 10-MV beams resulted in the highest neutron dose per MU with comparable values between CONV and SIB treatment plans. For 10-MV beams, omission of the flattening filter yielded 20% less neutron dose per fraction. The 10-MV SIB plans (flattened and FFF altogether) resulted over their entire treatment course (20 fx) in 38.17% (Table 3) decreased absolute neutron dose when compared to their CONV counterparts (37 fx). A negligible neutron dose (< 0.03 µSv/fx) was measured with 6‑MV treatment plans. When summed up over an entire treatment course of 37 treatment fx, 10-MV FLAT beams resulted in 13.1 mSv neutron dose vs. 10.9 mSv for FFF beam dose delivery (0.5 and 0.9 mSv for 6‑MV equivalents). A total of 20 fx of respective SIB plans resulted in 8.4 mSv neutron dose for 10-MV FLAT beams and 6.5 mSv for 10-MV FFF beams (0.3 and 0.6 mSv for their 6‑MV counterparts, Table 3).

**Table 3:** Difference in percent for the comparison between conventional (74 Gy) and simultaneous integrated boost (SIB 50/60 Gy) treatment concepts (left side) as well for comparison between FLAT and FFF plans (right side) in terms of resulting neutron doses (H_PN_) and out-of-field doses (D_OOF_) at respective nominal accelerator potentials (6 and 10 MV)

Effect SIB				Effect FFF			
Groups	Nom. acc. potential	H_PN_ (mSv)	D_OOF_ (mGy)	Groups	Nom. acc. potential	H_PN_ (mSv)	D_OOF_ (mGy)
6‑MV FLAT 74 Gy	6 MV	0.5 ± 0.1	59.2 ± 11.1	6‑MV FLAT 74 Gy	6 MV	0.5 ± 0.1	59.2 ± 11.1
6‑MV FLAT SIB 50/60 Gy	6 MV	0.3 ± 0.1	38.0 ± 10.0	6‑MV FFF 74 Gy	6 MV	0.9 ± 0.1	25.9 ± 3.7
Difference (%)		−34.74	−36.13	Difference (%)		104.08	−59.13
6‑MV FFF 74 Gy	6 MV	0.9 ± 0.1	25.9 ± 3.7	6‑MV FLAT SIB 50/60 Gy	6 MV	0.3 ± 0.1	38.0 ± 10.0
6‑MV FFF SIB 50/60 Gy	6 MV	0.6 ± 0.2	14.0 ± 4.0	6‑MV FFF SIB 50/60 Gy	6 MV	0.6 ± 0.2	14.0 ± 4.0
Difference (%)		−37.13	−39.85	Difference (%)		96.59	−61.51
10-MV FLAT 74 Gy	10 MV	13.1 ± 1.5	66.6 ± 14.8	10-MV FLAT 74 Gy	10 MV	13.1 ± 1.5	66.6 ± 14.8
10-MV FLAT SIB 50/60 Gy	10 MV	8.4 ± 1.5	40.0 ± 8.0	10-MV FFF 74 Gy	10 MV	10.9 ± 2.1	25.9 ± 7.4
Difference (%)		−36.10	−40.90	Difference (%)		−17.18	−60.72
10-MV FFF 74 Gy	10 MV	10.9 ± 2.1	25.9 ± 7.4	10-MV FLAT SIB 50/60 Gy	10 MV	8.6 ± 1.5	40.0 ± 8.0
10-MV FFF SIB 50/60 Gy	10 MV	6.5 ± 1.4	16.0 ± 4.0	10-MV FFF SIB 50/60 Gy	10 MV	6.5 ± 1.4	16.0 ± 4.0
Difference (%)		−40.23	−38.79	Difference (%)		−22.53	−59.31

A strong positive correlation between MUs and H_PN_ exists for all delivery scenarios with Pearson’s correlation coefficients over *r* = 0.93 (Table 2). The resulting data do not allow for differentiation between SIB and CONV within one standard deviation for all given scenarios. Weighted equivalent square field size (A_eq_) calculations show a moderate-to-strong negative correlation between H_PN_ and A_eq_ with Pearson’s correlation coefficients between r = −0.55 and −0.83 (Table 2). Therefore, neutron contamination depends obviously predominantly on beam energy and number of delivered MUs. Implementation of an SIB by itself does not result in markedly different neutron doses in comparison to CONV when MUs remain comparable.

### Distant out-of-field-dose in SIB and CONV plans with and without flattening filter, as a function of monitor units, and equivalent square field size

Out-of-field doses were lowest for all FFF scenarios (0.7 mGy/fx). The 6‑MV FLAT beams yielded higher doses (1.8 mGy/fx) and 10-MV FLAT resulted in the highest doses (1.9 mGy/fx). Within one standard deviation, CONV and the corresponding SIB plans were identical for all scenarios when a single treatment fraction was compared. Pearson’s correlation coefficients between MUs and D_OOF_ exhibited a strong positive correlation in all cases over *r* = 0.73, but was the highest for FLAT SIB 6‑MV and 10-MV treatment plans with *r* > 0.90. No clear correlation was found for the relationship between D_OOF_ and A_eq_.

In view of a complete treatment course, SIB plans yielded markedly lower out-of-field doses when compared with their respective CONV plans. A comparison between effects exerted from FFF-VMAT and SIB-VMAT resulted in a comparable reduction of D_OOF._ For example, for 10-MV FLAT VMAT, 60 Gy SIB RT resulted in 40.9% decreased D_OOF_ (compared with CONV RT) while FFF-mode led to 60.7% (CONV) and 59.3% (SIB) less D_OOF_ readings when compared to their referring FLAT counterparts (10-MV FLAT CONV, 10-MV FLAT SIB). The D_OOF_-sparing effect of SIB plans was similar for flattened and FFF plans (Table 3).

### Comparison of integrated effects of photon energy, SIB and flattening filter-free VMAT on photoneutron dose and distant out-of-field dose

As expected, for H_PN_ readings, photon energy exerted the greatest effect (F value 1647, *p* < 0.0001), while the influence of SIB was significantly smaller (F value 114, *p* < 0.0001) as was the effect of FFF-RT (F value 13.9, *p* = 0.0002).

Interactions were noted between FFF-RT and photon energy (F value 70.6, *p* < 0.0001) but also for SIB and photon energy (F value 215, *p* < 0.0001). Therefore, the influence of photon energy on H_PN_ is not consistent but depends on filter position and homogeneity. For the reference group 10-MV FLAT CONV, significantly higher H_PN_ readings were noted in comparison to the most promising plan setting 6‑MV FFF SIB (13.1 ± 1.5 mSv vs. 0.6 ± 0.2 mSv, estimate 12.5 ± 0.3 mSv, *p* < 0.0001) as opposed to slightly decreased H_PN_ readings for 6‑MV FLAT CONV when compared to 6‑MV FFF SIB (0.5 ± 0.1 mSv vs. 0.6 ± 0.2 mSv, estimate 0.13 ± 0.03 mSv, *p* < 0.0001, Table 4).

**Table 4:** Estimated marginal means (emmean) for neutron doses (H_PN_) and out-of-field doses (D_OOF_), their standard errors (SE), and confidence levels (CL) for the comparison of standard reference groups with the most promising plan setting (6-MV FFF SIB)

	10-MV Flat CONV vs. 6‑MV FFF SIB					
	Emmean	SE	Lower 95% CL	Upper 95% CL	F value	*p*
H_PN_ (mSv)	12.5	0.3	11.9	13.1	1688.6	< 0.0001
D_OOF_ (mGy)	50.8	2.8	45.7	55.9	328.1	< 0.0001
	6‑MV Flat CONV vs. 6‑MV FFF SIB					
	Emmean	SE	Lower 95% CL	Upper 95% CL	F value	*p*
H_PN_ (mSv)	−0.13	0.03	−0.18	−0.8	17.9	< 0.0001
D_OOF_ (mGy)	45.2	2.2	41.6	55.9	437.2	< 0.0001

F value indicates effect size, significance level *p*< 0.05

D_OOF_ readings were primarily influenced by the position of the FF (F value 1082, *p* < 0.0001) and to a smaller extent by SIB (F value 326, *p* < 0.0001) and even less by photon energy (F value 11, *p* = 0.001). An interaction was noted only between FFF-RT and SIB (F value 65.5, *p* < 0.0001). A comparison between 10-MV FLAT CONV and 6‑MV FFF SIB yielded significantly decreased D_OOF_ readings for the FFF SIB plan (66.6 ± 14.8 mGy vs. 14.0 ± 4.0 mGy, estimate 50.8 ± 2.8 mGy, *p* < 0.0001) as did the comparison between 6‑MV FLAT CONV and 6‑MV FFF SIB (59.2 ± 11.1 mGy vs. 14.0 ± 4.0 mGy, estimate 45.2 ± 2.2 mGy, *p* < 0.0001, Table 4).

## Discussion

This study is aimed to investigate whether implementation of a simultaneous integrated boost (SIB) results in an effect on neutron or scattered photon doses, possibly due to increased MLC activity, which has been identified as one primary source for neutron generation [10]. Furthermore, we explored whether the FFF mode may influence such an effect, if present.

In our 240 treatment sequences, the following case-dependent results were found: In 115 (for D_OOF_) and 118 (for H_PN_) out of 120 treatment courses, implementation of an SIB resulted in less D_OOF_ and H_PN_ when compared to CONV. The FFF mode was preferable over FLAT in all cases with respect to D_OOF_. For 6 MV, neutron doses were higher for FFF beams in all cases (60/60). For 10 MV, neutron doses were lower in 54 of 60 cases with the FFF mode. As expected, neutron doses were always higher for 10 MV than for 6 MV. On the other hand, D_OOF_ values were lower in 104 of the 120 cases for 6 MV.

The SIB concept leads to an increased fractional RT dose within a selected volume. When calculated for comparable efficacy, the EQD_2Gy_ model calculates for a given alpha/beta value a reduced total RT dose, which in turn implies a reduction in the fraction number, therefore shortening the overall treatment time. We found that our SIB scenario (2.5 Gy/3.0 Gy to 50 Gy/60 Gy) results in decreased total neutron dose as well as D_OOF_ when compared to conventional (CONV) fractionated 2 Gy to 74 Gy. Therefore, due to the higher fractional doses and thus reduced number of treatment fractions, an SIB is clearly preferable in this context. Flattening filter-free VMAT, effective in sparing both neutron dose (in 10-MV beams) and D_OOF_, respectively, increased this effect.

Omission of the flattening filter (FFF) significantly decreases both the photoneutron as well as the out-of-field dose in VMAT for prostate cancer at distant locations, even though FFF plans typically require more MUs than their flattened counterparts to compensate for the non-flatness of its profiles. Monitor units as surrogate for the modulation degree have a strong linear correlation with the formation of photoneutrons. By contrast, with respect to out-of-field dose, this strong linear correlation with MUs was only noted in flattened SIB plans.

We show that no increased neutron (H_PN_) or scattered photon dose (D_OOF_) results from increasing the fractional dose from 2.0 Gy to 2.5/3.0 Gy. Therefore, implementation of an SIB in a hypofractionated dose concept leads to less neutron and out-of-field dose in a complete treatment course and this effect was as distinct as the neutron dose-sparing and scattered radiation-sparing effect exerted by FFF VMAT.

Since the measurement position was kept constant at all times at 61.8 cm away from the beam isocenter, we were unable to detect a relationship between the distance to the radiation field and scattered dose. Hauri et al. [22] demonstrated a dependency of scattered dose on the distance from the isocenter. Between 40 and 80 cm away from the isocenter, total doses decrease slowly and the resulting scatter dose results mainly from collimator scatter and head leakage (and less from patient scatter). Haelg et al. [23] showed that neutron contamination depends only weakly on distance from the radiation field. Thus, a comparison between different treatment plan parameters (6 vs. 10 MV, FFF vs. FLAT, and SIB vs. CONV) appears to be appropriate at a fixed distance and the results presented may be used to approximate a whole-body exposure.

In our scenarios the boost dose is 3.0 Gy while the surrounding PTV receives 2.5 Gy, hence a ratio of 1.25 to 1.5 when compared to CONV treatment plans (2 Gy). For the sake of comparison, all absolute neutron and out-of-field dose readings were taken in relation with MUs to account for these differing dose levels. When analyzing all 30 patients, we found both increased and decreased numbers of MUs for the respective SIB plans compared to their corresponding CONV treatment plans. When the 120 SIB treatment plans were corrected by 3 Gy/2.5 Gy (to yield MU per Gray PTV dose), 57 SIB treatment plans had more MUs and 63 SIB plans exhibited less MUs than their CONV counterparts. Non-uniform target coverage therefore requires in every second case less MUs than their uniform equivalents. This also implies less neutron dose.

Due to the higher number of MUs required in FFF dose deliveries, the reduced neutron generation has to be set in relation to the difference in MUs to judge whether FFF or FLAT deliveries generate less neutron dose to the patient. In flattened SIB plans (6 MV and 10 MV), we noticed a comparable correlation between MUs and D_OOF_, an effect which was circumvented by the omission of the FF.

In AAPM report 158 [12], deposited neutron dose equivalents per MU for 6‑MV and 10-MV Elekta linacs are not provided. However, our finding of 0.5 µSv/MU for 10-MV FLAT are in line with the reported Varian 10-MV FLAT beam results of 0.9 µSv/MU and 0.3 µSv/MU for Siemens linacs. It is, moreover, noted in AAPM report 158 that Varian linacs possess a roughly twofold higher neutron contamination than comparable Elekta linacs.

Even though no relevant neutron dose was measured in 6‑MV beams, a stable signal was obtained at a very low level, which also showed a correlation between MUs and H_PN_ with *r* values between 0.93 and 1.0 (Table 2), and a slight increase in average neutron dose was noted for 6‑MV FFF when compared with 6‑MV FLAT. Of note, even for this beam energy, a reduction in H_PN_ is induced for an overall RT course with implementation of SIB (34.7 and 37.1% for 6‑MV FLAT and 6‑MV FFF, respectively; Table 3).

At the nominal energy of 6‑MV FLAT, the flattening filter does not contribute to PN contamination, because the bremsstrahlung is filtered with a stainless steel disc [24] and energy thresholds for PN production (γ, *n*) for iron and copper are 11.2 MeV (91.7%, Fe-56), 10.9 MeV (69.2%, Cu-63), and 9.9 MeV (30.3%, Cu-65; [25]). The same IAEA report lists all energy thresholds for PN production (γ, *n*) for the respective isotope and its natural abundance for tungsten. These were reported to be 8.1 MeV (26.3%, W‑182), 7.4 MeV (30.7% W‑184), 6.2 MeV (14.3%, W‑183), and 5.8 MeV (28.6%, W‑186). The 6‑MV Elekta linac’s maximum photon energies were reported to be 6.6 or 6.7 MeV, respectively [26, 27]. Furthermore, Elekta linacs—in contrast to Varian linacs—increase the initial electron energy for FFF beams up to a point where the D10cm of FLAT and FFF is identical (e.g., 67.5/73.5% of the maximum dose at 10 cm depth for 6/10 MV; [28]). This, in summary explains why detected Hpn values for 6‑MV FFF are higher than 6‑MV FLAT: 0.027 µSv/MU > 0.015 µSv/MU. For 10 MV this should apply as well; however, the flattening filter as an additional neutron source dominates and reverses the behavior 0.5 µSv/MU (10-MV FLAT) > 0.3 µSv/MU (10-MV FFF).

In our study, the SIB scenario reduced for 10-MV plans the total neutron dose from 13.1 to 8.4 mSv (36.1%) in flattened VMAT and from 10.9 to 6.5 mSv for FFF-VMAT (40.2%). FFF-VMAT reduced neutron dose from 13.1 mSV to 10.9 mSV (17.2%) for the conventional fractionated scenario and from 8.4 to 6.5 mSv (22.5%) for SIB-RT. Scattered radiation (D_OOF_) was likewise reduced for both 6‑MV and 10-MV beams with SIB by approximately 36.1–40.9% and even more effectively with FFF by approximately 59.1–61.5%. The lowest neutron and scattered radiation dose was derived with 6‑MV SIB (H_PN_ 0.3–0.6 mSv; D_OOF_ 14.0 mGy for 6‑MV FFF).

Treutwein et al. [29] compared the excess absolute risk (EAR) of secondary cancer in localized prostate IMRT with 6‑MV beams with and without flattening filter. For the secondary malignancy risk (SMR) in the periphery—corresponding to our distant measurement localization—they noted a reduced peripheral dose without flattening filter, which led to significantly reduced SMR. Murray et al. [13] described lower second cancer risks for FFF with increasing impact in organs at greater distance. This beneficial effect may be increased with SIB-RT. These reported SMR induced by scattered photons and electrons are very low for regions in the body at a distance from the primary treated region. Nevertheless, photoneutrons pose a significant risk in view of secondary cancers (ICRP 60; [30]) and even more so to implanted electronic devices because of their interaction with complementary metal oxide semiconductors and random access memory [31–33]. Hauri et al. presented data that showed that neutron dose equivalent remained unaffected by the distance from the isocenter and neutron dose peaked in the neck region along an axis from the pelvis (isocenter) to the head in an Alderson phantom, probably due to different thickness of penetrated tissue [22]. Corresponding to our data, Haelg et al. showed that neutron dose resulting from 15-MV IMRT produced by linacs from three different vendors (Elekta, Varian, and Siemens) was at least one order lower than the photon stray dose, therefore contributing with a very low amount to the total integral dose of a patient [23]. Still, regarding neutron dose, significant effects were noted in patients and phantom studies with respect to cardiac implanted electronic devices (CIEDs, [32, 33]). These devices are implanted near the neck region under the clavicular bone in a subcutaneous pouch, therefore in the peak region for neutron dose [22]. In a phantom study, 10-MV FFF-VMAT of the prostate resulted in pacing inhibition, data loss and reset in 4 of 15 ICDs that were located at a distance from the primary beam [19]. In an exemplary clinical survey, 10-MV FFF-VMAT resulted in an ICD error [32]. Other clinical studies demonstrate that photon beams >6 MV result in CIED errors [31–33] even though beam energies were widespread between 10 and 23 MV. International guidelines advocate the use of non-neutron-producing beam energies in CIED-bearing patients, but discrepancy exists between a lower beam energy threshold (6 vs. 10 MV, [18, 31, 33–35]). No data exist for the disaggregation between 10-MV FLAT and 10-MV FFF in regard to CIED effects even though we have shown a 20% reduction in neutron dose in 10-MV FFF VMAT when compared to their 10-MV FLAT counterpart plans (for the same fx dose level). Therefore, although we show that SIB can further reduce H_PN_ in FFF-VMAT (H_PN_ 10-MV FLAT > 10-MV FFF > 10-MV FLAT SIB > 10-MV FFF SIB), we cannot advise for the use of 10-MV FFF beams even for RT with SIB in CIED-bearing patients and have to wait for further research on this topic.

Our study has some limitations that need consideration. We only used one measurement point approximately 60 cm from the field edge 0.5 cm below the surface of the phantom. We chose this location with reference to Hauri et al. [22]. At 60 cm distance, there is almost exclusively head leakage contribution and almost no patient scatter. Thus, past this distance the measured doses remain approximately constant. Closer to the radiation field, both Hauri et al. and AAPM report 158 [12] report an approximately exponential decay of patient scatter and a constant head leakage contribution. Furthermore, this distance between detectors and radiation field reflects clinically the distance between prostate RT and CIEDs [19] and considers H_PN_ and D_OOF_ at their typical location in the body.

## Conclusion

In conclusion, implementation of a simultaneous integrated boost (SIB) and omission of the flattening filter (FFF) reduces neutron contamination and out-of-field doses per monitor unit for volumetric modulated arc therapy (VMAT) for prostate cancer. SIB treatments lead to lower neutron and out-of-field doses for an overall treatment course. Neutron contamination in VMAT for prostate cancer depends, as expected, predominantly on the beam energy and the number of delivered monitor units. Due to the higher number of monitor units typically required in flattening filter-free (FFF) dose deliveries with this particular set-up, the reduced neutron generation per monitor unit has to be set in relation to the difference in monitor units to judge whether FFF or FLAT deliveries generate a lower neutron and out-of-field dose to the patient. Respecting radiation protection principles, an implementation of an SIB in moderately hypofractionated prostate RT may be advisable not only for shortening of overall treatment time.
